# Is Maternal Carbohydrate Intake Having an Impact on Newborn Birth Weight? A Systematic Review

**DOI:** 10.3390/nu15071649

**Published:** 2023-03-28

**Authors:** Malshani L. Pathirathna, Hapugahapitiye M. R. K. G. Nandasena, Buddhini P. P. Samarasekara, Thakshila S. Dasanayake, Ishanka Weerasekara, Megumi Haruna

**Affiliations:** 1 Department of Midwifery and Women’s Health, Division of Health Sciences and Nursing, Graduate School of Medicine, The University of Tokyo, Tokyo 113-0033, Japan; malshanilakshika@ahs.pdn.ac.lk; 2 Department of Nursing, Faculty of Allied Health Sciences, University of Peradeniya, Peradeniya 20400, Sri Lanka; renukalhari@ahs.pdn.ac.lk (H.M.R.K.G.N.); sdbppsamarasekara@ahs.pdn.ac.lk (B.P.P.S.); 3 Ross and Carol Nese College of Nursing, The Pennsylvania State University, University Park, PA 16802, USA; tsd5277@psu.edu; 4 Department of Health and Functioning, Faculty of Health and Social Sciences, Western Norway University of Applied Sciences, 5063 Bergen, Norway; imwrm@hvl.no; 5 School of Health Sciences, The University of Newcastle, Callaghan, NSW 2308, Australia; 6 School of Allied Health Science and Practice, Faculty of Health and Medical Sciences, The University of Adelaide, Adelaide, SA 5005, Australia

**Keywords:** maternal nutrition, macronutrients, carbohydrate intake, birth weight, systematic review

## Abstract

Glucose is a vital fuel for fetal growth, and carbohydrates are the primary source of glucose in the diet. The effects of carbohydrate intake during pregnancy on neonatal birth weight have not been fully investigated or systematically reviewed. Therefore, this systematic review aimed to collate the available evidence to determine whether carbohydrate intake during pregnancy impacts newborn birth weight. A literature search was performed from inception to March 2022 in Embase, Medline, and PsycInfo. Articles published in English were independently screened for the title and abstracts, and then for full texts. Out of 17 studies included, a significant relationship between the intake of maternal carbohydrate or its subcomponents and neonatal birth weight was reported in six studies. Of them, one study reported that higher carbohydrate intake in early pregnancy was associated with lower birth weight. The two other studies reported a positive correlation between maternal carbohydrate intake and neonatal birth weight regarding first- and second-trimester intake. Maternal carbohydrate intake may have an impact on birth weight, as suggested by the included studies in this systematic review. However, the overall review indicates contradictory findings concerning the relationship between carbohydrate intake and neonatal birth weight. Studies assessing the type of carbohydrate and the amount consumed with improved methodological quality are recommended.

## 1. Introduction

Epidemiological evidence suggests that intrauterine and early-life exposures may impact adult diseases later in life [1,2]. The Development Origins of Health and Disease (DOHaD) theory proposes that some diseases in later life are formed during the fertilization, embryonic, fetal, and neonatal stages by the interrelation between genes and the environment. In this concept, adverse environmental factors caused by poor nutrition, infections, chemicals, and metabolite or hormonal perturbations [3] may cause changes in fetal growth and metabolisms to adapt to the environment; however, this process may become maladaptive and lead to disease later in life if the environment changes [4]. Studies evaluating the programming effect of intrauterine growth on various diseases have utilized birth weight as a proxy of intrauterine growth [5,6,7]. Hence, it is vital to identify the modifiable factors associated with neonatal birth weight.

Glucose is a vital fuel for fetal growth, is carried across the placenta by facilitated diffusion, and is dependent on the maternal–fetal concentration gradient. Gluconeogenesis is virtually absent during fetal life; hence, the fetus gets almost all of its glucose from the mother’s circulation. Thus, maternal glucose and other metabolic fuels provide energy for fetal growth. Maternal glucose is produced by the metabolism from endogenous sources and the maternal diet, mainly from carbohydrates.

Several local and international dietary guidelines have advised higher energy intake during pregnancy, but no particular recommendations have been made on the macronutrient composition of the maternal diet [8]. A number of studies have attempted to develop and test macronutrient and micronutrient dietary strategies to guarantee enough energy intake and specific nutrients to meet maternal and fetal needs [9]. These nutritional interventions include antenatal dietary counseling [10], balanced protein-energy supplementation (<25% of total energy from protein), high-protein diets (25% of the total energy is from protein), isocaloric protein supplements (protein replaces an equal amount of nonprotein energy), and low-energy diet recommendations for pregnant women who are overweight or who have rapid gestational weight gain in early pregnancy. Among these interventions, the data from several reviews suggest that balanced protein-energy supplementation is the most effective macronutrient intervention that leads to modest gestational weight gain and neonatal birth weight [11,12,13], along with a reduction in risk of small-for-gestational-age (SGA) newborns among undernourished women [14]. Moreover, a review of Cochrane systematic reviews and randomized controlled trials on humans suggested that calcium supplementation protects against low birth weight, and that magnesium supplementation protects against SGA [9].

However, the effects of maternal carbohydrate intake on neonatal birth weight have not been thoroughly investigated, even though it is the primary glucose source in the maternal diet. Moreover, there are discrepancies in the findings even though there have been several studies on maternal dietary components and birth size worldwide. Furthermore, maternal nutrition might be differently associated with neonatal birth weight in higher-income countries than in other countries because of the other associated factors [15]. Thus, we aimed to collate the available scientific evidence to determine whether carbohydrate intake during pregnancy impacts newborn birth weight.

## 2. Materials and Methods

### 2.1. Eligibility Criteria

Observational studies that investigated the association of carbohydrate intake during pregnancy with neonatal birth weight in human participants in any setting and published as peer-reviewed full articles were included in this systematic review. The studies not published in English and study designs such as case studies, case reports, commentaries, editorials, letters to the editor, reports, reviews, and systematic reviews were excluded.

### 2.2. Information Sources and Search Strategy

The study protocol was registered with the International Prospective Register of Systematic Reviews on 8 March 2022 (CRD42022298544). The literature search was conducted on Embase, Medline, and PsycInfo without restriction to a specific period to locate all eligible articles using words related to pregnancy, carbohydrates, newborns, and birth weight (Appendix A). The search was restricted to articles published in English. In addition, the reference lists of located articles were scanned to attain relevant additional articles.

### 2.3. Study Selection

The updated Preferred Reporting Items for Systematic Reviews and Meta-Analysis (PRISMA) guidelines were followed. The database search results were exported into the Endnote (EndNote X9. 3. 3 version) reference manager software, and duplicated studies were removed. The remaining studies’ titles and abstracts were screened against the inclusion and exclusion criteria to identify the potential full-texts by at least two independent reviewers (M.L.P., H.M.R.K.G.N., T.S.D., and B.P.P.S.) using the Covidence systematic review software (Veritas Health Innovation, Melbourne, Australia). Any discrepancies were resolved by discussion among reviewers. The same screening procedure was carried out for the full-text selection.

### 2.4. Data Extraction

The data from the included studies were extracted by one author (M.L.P.) and checked at least by one other author (H.M.R.K.G.N. and M.H.) for accuracy. The extracted data included the study characteristics (author/s, study period, country, setting, and study design), characteristics of the maternal and newborn pairs (sample size, age, pre-pregnancy BMI, parity, smoking and alcohol consumption during pregnancy, gestational age at delivery, and neonatal birth weight), exposure/s, outcome/s, trimester studied, confounders/covariates, findings related to carbohydrate intake and birth weight, methods of dietary assessment, and quantified dietary intakes (total energy intake, carbohydrate intake, and percentage of energy from carbohydrate).

### 2.5. Quality Appraisal

Two authors (M.L.P. and H.M.R.K.G.N.) independently assessed the quality of included studies using the National Institute of Health (NIH) study quality assessment tool for observational, cohort, and cross-sectional studies, as well as the NIH study quality assessment tool for case–control studies [16]. Consensus between the two assessors was used to resolve any disagreements.

### 2.6. Data Analysis

Descriptive statistics were used to report the results of the included studies. Tables and graphs were used appropriately to convey the publication information, participant characteristics, and summary findings according to the objectives of this systematic review.

## 3. Results

### 3.1. Study Selection

**Figure 1:**
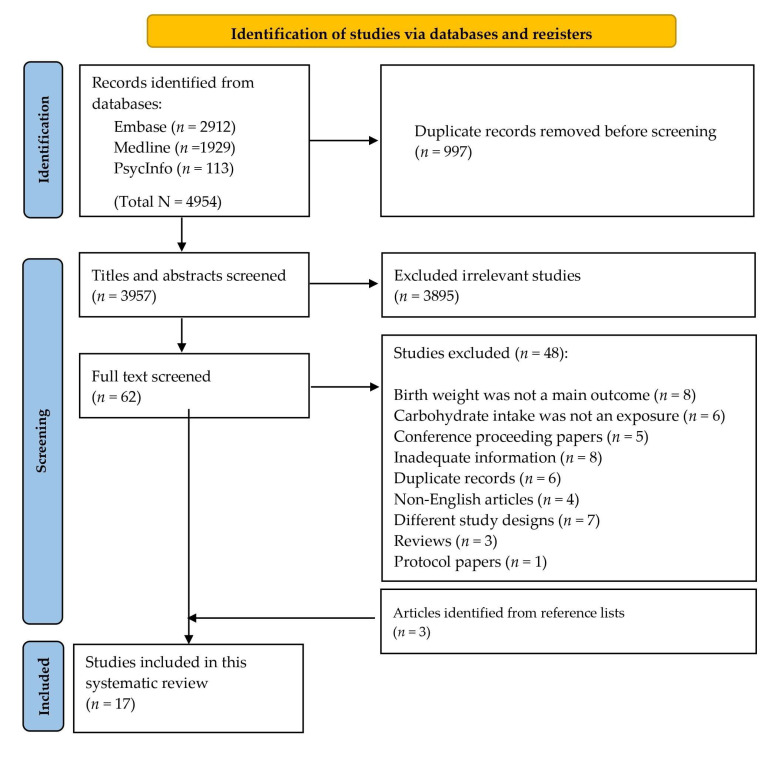
PRIMA flow chart for included studies.

The database search resulted in the identification of 3957 articles after the removal of duplicates. Of these, 62 articles were deemed potentially relevant following the title and abstract screening. On screening of the full text, another 48 articles were excluded. An additional three relevant full-texts were found by scanning the reference lists of the located articles. This left 17 articles [17,18,19,20,21,22,23,24,25,26,27,28,29,30,31,32,33] for inclusion in this review (Figure 1).

### 3.2. Characteristics of Included Studies

Out of the 17 included studies, there were four (23.5%) studies from the United Kingdom [17,18,19,30], three (17.6%) studies from the United States [20,25,28], two (11.7%) studies from Japan [32,33], and one each (5.9%) from Australia [21], Tunisia [22], Jordan [23], New Zealand [24], Germany [26], Sri Lanka [27], Malawi [29], and Spain [31]. Of the total, 13 studies (76.5%) utilized cohort designs [17,18,19,20,21,24,25,26,27,28,30,32,33], while the remaining studies followed cross-sectional [23,29] and case–control [22,31] designs (Table S1).

### 3.3. Characteristics of Study Participants

The sample size of included studies ranged from 41 to 78,793 maternal and newborn units. The reported youngest mean maternal age (25.8 ± 4.9 years) was from a study conducted in the United Kingdom [18]. Almost all the reported mean pre-pregnancy body mass index (BMI) values were within the limit of normal and overweight BMI categories according to the international classification of BMI [34]. The lowest mean neonatal birth weight (2874.6 ± 497 g) was reported from Sri Lanka [27], while the highest overall mean birth weight (3551.0 ± 544 g) was reported from New Zealand [24] (Table S2).

### 3.4. Reported Energy and Carbohydrate Intakes during Pregnancy

Table 1 shows pregnant women’s energy and carbohydrate consumption quantified in the included studies. Of the 17 studies, 13 reported [17,18,19,21,22,23,24,25,26,27,29,30,32] pregnant women’s total energy and carbohydrate consumption. Three studies [17,18,21] reported maternal dietary intake in early pregnancy. Among them, a study conducted in the United Kingdom [17] reported the highest median intake of total energy [2329 (1882, 2789) kcal/day] and carbohydrate [302.7 (245.7, 372.9) g/day] during early pregnancy. Three studies mainly focused on second-trimester maternal nutrition [26,27,30], whereas one study reported nutrient consumption during the fourth month of the pregnancy [24]. Out of these four studies, the Sri Lankan study reported the highest mean intake of total energy (2921.5 kcal/day) and carbohydrates (532.7 g/day) during the second trimester of pregnancy. A total of eight studies investigated maternal nutrition during the third trimester/late pregnancy/at the seventh month of pregnancy [17,18,19,21,24,26,28,29] with the highest median carbohydrate intake of 377.0 g/day [29]. Only five studies [17,18,19,21,25,31] reported the percentage of energy derived from carbohydrates, and its median ranged from 47.3% to 55.3%.

### 3.5. Summary of Findings Related to Carbohydrate Intake and Neonatal Birth Weight

More than 50% of the studies included in this review utilized a food frequency questionnaire (FFQ) [17,18,20,21,22,23,27,31,32], while three studies used food diaries alone [19] or in combination with FFQ [18] or 24 h dietary recall (24 HDR) [24]. A total of five studies utilized only 24 HDR [25,26,28,29,30].

Among the 17 studies, a significant relationship between maternal carbohydrate intake or its subcomponents and neonatal birth weight was reported only in six studies [17,24,27,30,31,33]. Of them, one study reported that higher carbohydrate intake in early pregnancy was associated with lower birth weight [17]. In contrast, the other two studies reported a positive correlation between maternal carbohydrate intake and neonatal birth weight regarding first-trimester [30] and second-trimester intake [27]. A study conducted in New Zealand reported a quadratic relationship between birth weight and the percentage of total energy from carbohydrates, fat, and protein throughout pregnancy, indicating that birth weight was greatest when the percentage of total energy from carbohydrates, fat, and protein was 48%, 35%, and 17%, respectively [24]. Only one study utilized the brief self-administered diet history questionnaire (BDHQ), suggesting that more extensive changes in sucrose consumption from the first to the second trimester of pregnancy are associated with the delivery of babies with birth weight ≥90th percentile [33]. One study focused on the type of carbohydrates consumed by the mothers. It revealed that consumption of more than 75 g/day of brown bread was inversely associated with delivering babies with a birth weight of <10th percentile, while consumption of industrial sweets more than once a day or even 2–6 times a week increased the risk for same [31] (Table 2).

### 3.6. Other Findings Related to Nutrient Intake and Neonatal Birth Weight

Three studies reported a positive association between maternal vitamin C intake and neonatal birth weight [18,29,32], while two reported a similar association with maternal vitamin A intake [23,32]. Moreover, one study revealed that early pregnancy protein-energy content positively correlated with neonatal birth weight [21]. At the same time, neonatal birth weight was inversely correlated with maternal fat consumption and intake of polyunsaturated fatty acids (PUFAs) in early pregnancy [30] (Table 2).

### 3.7. Quality Appraisal of Included Studies

**Figure 2:**
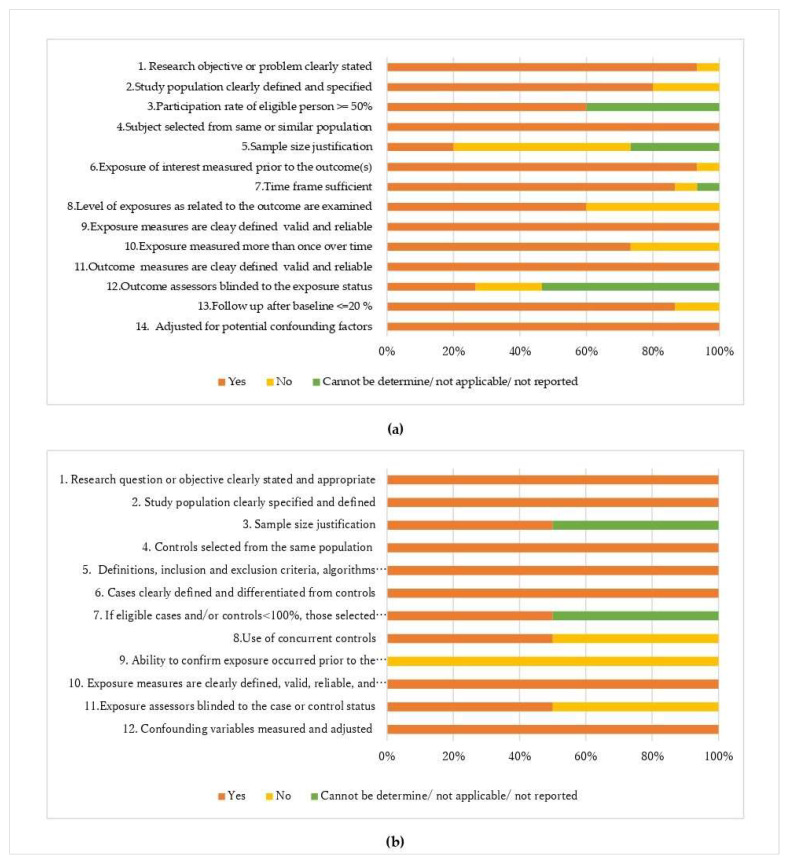
Quality assessment of included (**a**) cohort and cross-sectional studies (*n* = 15 studies), and (**b**) case–control studies (*n* = 2 studies) using the National Institute of Health (NIH) study quality assessment tools [16].

A clearly stated research question or objective was specified in nearly all studies (94.1%) included in this systematic review, and 82.4% of the studies clearly defined their study population. Moreover, 60% of the cohort and cross-sectional studies had an adequate (i.e., >50%) subject participation rate representing the eligible subject. 76.5% of the cohort and cross-sectional studies reported an appropriate exposure time frame. The two main issues for all the included studies were the blinding of the assessors to the exposure status (29.4%) and the availability of a sample size rationale (23.5%). The criteria for adjusting for potential confounding factors were met by each of the studies included in this review (Figure 2a,b).

## 4. Discussion

To the best of our knowledge, this is the first study to collate published studies to systematically explore the relationship between carbohydrate intake in pregnancy and neonatal birth weight. This systematic review presents findings based on 86,461 maternal and newborn pairs. In this comprehensive literature from 13 (76.5%) developed countries [17,18,19,20,21,24,25,26,28,30,31,32,33] and four developing countries [22,23,27,29], we found no relationship between carbohydrate intake and neonatal birth weight in 64.7% of the included studies. However, some studies reported relationships between neonatal birth weight and carbohydrate intake [17,27,30], the proportion of energy from carbohydrate intake [24], and the sub-components of carbohydrate intake [31,33]. Among these, a study conducted by Godfrey et al. in the United Kingdom suggested an inverse relationship between early pregnancy maternal carbohydrate intake and neonatal birth weight [17]. In contrast, a study conducted by Sharma et al. in the United Kingdom about two decades after Godfrey’s study found a positive correlation between first-trimester maternal carbohydrate intake and neonatal birth weight, as well as between glucose and lactose intake in pregnancy second trimester and the neonatal birth weight [30]. However, the different findings of the two studies may be attributable to the different methods employed in the two studies, as Sharma et al. utilized a 24 HDR, whereas Godfrey et al. utilized an FFQ. Moreover, Sharma et al. adjusted their findings for maternal smoking and alcohol consumption [30], while Godfrey et al. adjusted for gestational age at delivery and the gender of the newborn [17]. A study conducted in Sri Lanka, a developing country in South Asia, also found a positive association between second-trimester maternal carbohydrate intake and neonatal birth weight [27].

Furthermore, a study conducted in Japan revealed that more extensive changes in sucrose consumption from the pregnancy first to second trimester have an association with delivering large-for-gestational-age (LGA) babies (birth weight ≥90th percentile) [33]. None of the studies found any relationship between late-pregnancy carbohydrate intake and neonatal birth weight. A possible explanation might be that placentation is established, and fetal growth is programmed in the first trimester [35,36]. It is biologically plausible that nutritional effects on the fetus could vary with the time of pregnancy because fetal development and nutrient needs are structured over time [21].

Despite our hypothesis that maternal carbohydrate intake should positively correlate with newborn birth weight because it is one of the major sources of glucose and the primary energy source for fetuses, the studies compiled for this systematic review revealed varying results. These differences may be because not all carbohydrates affect blood sugar levels similarly. The type of carbohydrate taken determines the glycemic response to it. The glycemic index (GI) is a qualitative measure used to categorize different types of carbohydrates based on the metabolic reaction they cause. While carbohydrates with a lower GI index break down more steadily and boost blood glucose levels gradually, those with a higher GI index are quickly absorbed and can cause a sudden rise in blood glucose levels [37]. This is why mothers with gestational diabetes mellitus are recommended to consume a low-GI diet that reduces the risk of fetal macrosomia and other adverse pregnancy outcomes. In addition, the GI for food may also be influenced by the food processing and preparation method. Moreover, the glucose response depends upon the amount of carbohydrates and the type. Furthermore, the retrospective study design of some of the included studies might have caused recall bias when reporting dietary intakes, and the utilization of different dietary assessment methods could have led to the over- or underestimation of actual nutritional intake.

It has been argued that maternal nutrient intakes have a more significant impact on birth outcomes in less affluent populations [18], but this did not appear true for this systematic review with regard to maternal carbohydrate intake.

### Strengths and Limitations

This is the first systematic review to assess the impact of maternal carbohydrate intake during pregnancy on neonatal birth weight. There were several strengths of this review. These include the comprehensive search strategy and the eligibility criteria we used to retrieve all kinds of studies addressing the research objectives. This review provides a rigorous view of current evidence based on three databases. Furthermore, all studies included in this systematic review were adjusted for confounding, which is essential in causal observational studies.

The current systematic review also had some limitations to acknowledge. The language of the review was limited to English only. This may have led to the exclusion of some publications written in other languages. More data were needed to analyze the studies quantitatively; therefore, a meta-analysis could not be conducted. Furthermore, many studies in this review were collated from developed countries with only meager contributions from middle- and low-resource countries. Additionally, we could not evaluate other perinatal outcomes in this review, such as gestational diabetes mellitus and gestational weight gain, with respect to maternal nutrient intake, due to a lack of sufficient data in the included studies.

## 5. Conclusions

Carbohydrate is a primary maternal glucose source, providing energy and nutrients for the growing fetus. The present systematic review indicated contradictory findings concerning the relationship between carbohydrate intake and neonatal birth weight. This may have been because the included studies focused on either the type or the amount of carbohydrates consumed, whereas the glucose response depends on both the type and the amount of carbohydrates. Therefore, studies assessing the type of carbohydrate or GI and the amount consumed with improved methodological quality would help to analyze the impact of carbohydrates on neonatal birth weight in more detail.

## Figures and Tables

**Table 1:** Energy and carbohydrate consumption among pregnant women.

Study	Energy Intake (kcal/day) ^a^	Carbohydrate Intake (g/day) ^a^	% of Energy Derived from Carbohydrates ^a^
Godfrey et al., 1996 [17]	EP: 2329 (1882, 2789); LP: 2314 (1970, 2729)	EP: 302.7 (245.7, 372.9); LP: 301.9 (254.3, 360.6)	EP: 49.4 (46.2, 53.4); LP: 49.0 (46.1, 52.2)
Mathews et al., 1999 [18]	EP: 2044 (1755, 2305); LP: 2197 (1824, 2660)	EP: 256.0 (218.0, 292.5); LP: 290.1 (242.5, 350.5)	EP: 47.3 (44.1, 50.4); LP: 50.1 (46.1, 53.7)
Langley-Evans et al., 2003 [19]	FT: 2008 (1794, 2312); TT: 2023 (1802, 2279)	Carbohydrate: FT: 262.4 (228.3, 299.6); TT: 272.7 (232.3, 302.2), Sugar: FT: 15.2 (90.3, 142.1); TT: 116.7 (93.7, 150.4), Starch: FT: 143.9 (123.0, 168.0); TT: 143.5 (122.4, 162.1)	FT: 49.0 (45.0, 52.7); TT: 48.7 (46.2, 52.5)
Lagiou et al., 2004 [20]	NR	NR	NR
Moore et al., 2004 [21]	EP: 2151.1 (1720.8, 2772.5); LP: 2198.9 (1792.5, 2700.8)	EP: 273 (213, 349); LP: 282 (230, 347)	EP: 48.3 (44.5, 53.5); LP: 49.1 (45.0, 53.3)
Denguezli et al., 2009 ^b^ [22]	Total: 2701.0 ± 622.0; CAs: 3124.9 ± 403.0;COs: 2626.9 ± 624.6	Total: 413.6 ± 105.7; CAs: NR;COs: NR	NR
Bawadi et al., 2010 ^c^ [23]	2603.7 ± 32.9	320.8 ± 3.8	NR
Watson and McDonald, 2010 ^d^ [24]	4th month: 2261.2; 7th month: 2199.8	Carbohydrate: 4th month: 270; 7th month: 267, Glucose: 4th month: 26; 7th month: 26, Fructose: 4th month: 27; 7th month: 27, Sucrose: 4th month: 61; 7th month: 63, Lactose:4th month: 17; 7th month: 18, Maltose: 4th month: 3; 7th month: 3, Starch: 4th month: 136; 7th month: 129	NR
Crume et al., 2016 [25]	2025.0 (1789.6, 2252.6)	242.3 (195.6, 291.6)	47.8 (42.3, 53.2)
Diemert et al., 2016 ^b^ [26]	FT: 1987 ± 505; ST: 2068 ± 463;TT: 2151 ± 472	FT: 239 ± 65; ST: 245 ± 66;TT: 254 ± 66	49%
Pathirathna et al., 2017 ^b^ [27]	ST: 2921.5 ± 687.7	ST: 532.7 ± 133.8	NR
Grandy et al., 2018 ^b^ [28]	TT: 2382 ± 556	NR	51 ± 8
Hjertholm et al., 2018 [29]	TT: 2096.5 (1778.1, 2570.6)	TT: 377 (306, 454)	NR
Sharma et al., 2018 ^b^ [30]	FT: 2120 ± 692;ST: 2279 ± 634	Carbohydrate: FT: 274 ± 99; ST: 300 ± 92, Added sugar: FT: 127 ± 73; ST: 149 ± 69, Glucose: FT: 25 ± 19; ST: 27 ± 17, Fructose: FT: 27 ± 28; ST: 30 ± 24, Sucrose: FT: 54 ± 36; ST: 64 ± 36, Maltose: FT: 2 ± 7; ST: 2 ± 3, Lactose: FT: 16 ± 13; ST: 19 ± 15,	NR
Amezcua-Prieto et al., 2019 [31]	NR	NR	NR
Eshak et al., 2020 [32]	1620 (1311–2015)	223.8 (182.6, 272.4)	55.3 (50.2, 60.3)
Minato-Inokawa et al., 2020 ^b^ [33]	SGA group: 1444 ± 452; AGA group: 1500 ± 465; LGA group: 1506 ± 482	Carbohydrate ^b,e^: SGA group: 144.6 ± 23.1; AGA group: 143.0 ± 18.3; LGA group: 145.0 ± 9.6, Sucrose ^b,e^: SGA group: 6.9 ± 3.4; AGA group: 6.4 ± 4.3; LGA group: 6.3 ± 5.9	NR

^a^ Median (lower quartile, upper quartile) if not mentioned otherwise. Values are expressed as the ^b^ mean ± standard deviation, ^c^ mean ± standard error, and ^d^ means in ^e^ grams per 1000 kcal/day AGA: appropriate-for-gestational-age; CAs: cases; COs: controls; EP: early pregnancy; FT: first trimester; LGA: large-for-gestational-age; LP: late pregnancy, NR: not reported; SGA: small-for-gestational-age; ST: second trimester; TT: third trimester.

**Table 2:** Summary of findings from the included studies.

Study	Trimester/Period Studied	Method of Dietary Assessment	Confounders/Covariates	Significant Findings Related to Carbohydrate Intake and Birth Weight	Significant Findings Related to Other Nutrient Components and Birth Weight
Godfrey et al., 1996 [17]	Early pregnancy and late pregnancy (Median durations 15.3 and 32.7 weeks)	FFQ	Gestational age at delivery; newborn gender	A higher intake of carbohydrates in early pregnancy was associated with lower birth weight, especially if combined with low dairy protein intake in late pregnancy.	None
Mathews et al., 1999 [18]	First trimester and third trimester	7 day food diary, FFQ	Maternal smoking, height, nutrient intake, gestational age at delivery, gender of the newborn	None	Birth weight was positively associated with early maternal intake of vitamin C.
Langley-Evans et al., 2003 [19]	First trimester and third trimester	5 day food diary	Social class based on partner’s occupation, maternal weight at booking, maternal smoking, gestational age at delivery, gender of the newborn	None	None
Lagiou et al., 2004 [20]	Second trimester	FFQ	Maternal age, height, pre-pregnancy BMI, education, parity, pre-gravid oral contraceptive use, maternal smoking, gestational age at delivery, gender of the newborn	None	None
Moore et al., 2004 [21]	Early pregnancy and late pregnancy (before 16 weeks and 30–34 weeks gestation)	FFQ	Maternal smoking, alcohol consumption, height, pre-pregnancy weight, parity, use of recreational drugs, gestational age at delivery	None	The percentage of energy derived from protein in early pregnancy was positively associated with birth weight.
Denguezli et al., 2009 [22]	Third trimester: the last 24 h before delivery	FFQ	Pre-pregnancy BMI, parity, term	None	None
Bawadi et al., 2010 [23]	Retrospectively collected data during 1–2 days postpartum focusing on the dietary intake during gestation	FFQ	Maternal pre-pregnancy BMI, parity, gestational weight gain	None	Birth weight was positively associated with maternal calcium and vitamin A intake.
Watson and McDonald, 2010 [24]	Month 4 and month 7 of pregnancy	24 HDR, 3 day food diary	Maternal height, weight, smoking, number of preschoolers, number of other adults in the house, gestational age at delivery (modified for over term babies), gender of the newborn	A quadratic relationship was established between birth weight and the percentage of total energy from carbohydrates, fat, and protein throughout pregnancy; birth weight was greatest when the percentage of total energy from carbohydrates was 48%, along with 35% fat and 17% protein.	Birth weight was greatest when the percentage of total energy from carbohydrates was 48%, along with 35% fat and 17% protein.
Crume et al., 2016 [25]	Throughout pregnancy	24 HDR	Maternal age, pre-pregnancy BMI, gravidity, race/ethnicity, smoking at any time during pregnancy, postnatal age at air displacement plethysmography measurement, physical activity levels during pregnancy, gestational age at delivery, gender of the newborn	None	None
Diemert et al., 2016 [26]	First trimester (12 + 0 to 14 + 6 weeks); second trimester (22 + 0 to 24 + 6 weeks); third trimester (34 + 0 to 36 + 6 weeks gestation)	24 HDR	Maternal age, pre-pregnancy BMI, level of education, gestational age at delivery	None	None
Pathirathna et al., 2017 [27]	Second trimester	FFQ	Gestational age at delivery, average monthly income, area of residence, history of low-birth-weight delivery	The newborns of women with low carbohydrate intake during pregnancy second trimester were lighter than those of women with a moderate carbohydrate intake.	None
Grandy et al., 2018 [28]	Third trimester (37–38 weeks gestation)	24 HDR	Maternal age, pre-pregnancy BMI, parity, total energy intake	None	None
Hjertholm et al., 2018 [29]	Third trimester	3 day repeated interactive multi-pass 24 HDR, 4 day repeated single-pass 24 HDR	Maternal age, weight, height, gestational age at delivery, literacy, marital status, household assets, parity, total energy intake, gender of the newborn	None	Birth weight was positively associated with vitamin C and the frequency of milk intake.
Sharma et al., 2018 [30]	First trimester, second trimester	24 HDR	Alcohol and smoking consumption status	The first-trimester maternal carbohydrate intake was positively associated with neonatal birth weight, while second-trimester maternal glucose and lactose intake were positively associated with neonatal birth weight.	Birth weight was negatively associated with early pregnancy maternal fat consumption and higher intake of PUFAs.
Amezcua-Prieto et al., 2019 [31]	Retrospective collection of data during 1–2 days of postpartum focusing on the dietary intake during the previous year	FFQ	Maternal level of education, pre-pregnancy BMI, parity, history of low-birth-weight delivery, history of preterm delivery, total energy intake, maternal smoking	Consumption of brown bread >75 g/day was inversely correlated with SGA, while industrial sweets more than once a day or even 2–6 times a week increased the risk of SGA births.	None
Eshak et al., 2020 [32]	Throughout pregnancy	FFQ	Maternal age, height, pre-pregnancy BMI, education, parity, household income, use of folate supplements, maternal smoking, alcohol consumption, gestational weight gain, gestational age at delivery, gender of the newborn	None	Birth weight was positively associated with maternal intake of total energy, dietary fiber, folate. and vitamins A, K, E, D, and C.
Minato-Inokawa et al., 2020 [33]	First trimester, second trimester, third trimester	BDHQ	Total energy intake	Mothers with LGA showed larger changes in plant oil and sucrose consumption from the first to the second trimester of pregnancy.	None

BDHQ: brief-type self-administered diet history questionnaire; BMI: body mass index; FFQ: food frequency questionnaire; LGA: large-for-gestational-age; PUFAs: polyunsaturated fatty acids; SGA: small-for-gestational-age; 24 HDR: 24 h dietary recall.

## Data Availability

All relevant data are included in the paper and the Supplementary Materials.

## Supplementary Materials

The following supporting information can be downloaded at https://www.mdpi.com/article/10.3390/nu15071649/s1: Table S1. Characteristics of included studies, ordered by year of publication; Table S2. Characteristics of study participants.

## Appendix A

Medline search strategy used in the systematic review to determine whether maternal carbohydrate intake has an impact on newborn birth weight.

	Search Items
1	matern*.mp. or pregnancy/
2	mothers/or mother*.mp.
3	pregnan*.mp.
4	(maternal adj3 carbohydrate*).mp. [mp = title, abstract, original title, name of substance word, subject heading word, floating sub-heading word, keyword heading word, organism supplementary concept word, rare disease supplementary conceptword, unique identifier, synonyms]
5	1 OR 2 OR 3 OR 4
6	dietary carbohydrates/or CHO.mp.
7	carbohydrates/
8	starch.mp. or starch/
9	sugar.mp. or sugars/
10	6 OR 7 OR 8 OR 9
11	infant, newborn/or*.mp.
12	infant/or infant*.mp.
13	(baby or babies).mp.
14	birth weight/or birth?weight.mp
15	(birth?weight adj5 (neonate* or infant* or baby or babies or newborn*)).mp.[mp=title, abstract, original title, name of substance word, subject heading word, floating sub- heading word, keyword heading word, organism supplementary concept word, raredisease supplementary concept word, unique identifier, synonyms]
16	pregnancy outcomes/or pregnancy outcome*.mp.
17	11 OR 12 OR 13 OR 14 OR 15 OR 16
18	5 AND 10 AND 17
