# Supplementary material for: Is Maternal Carbohydrate Intake Having an Impact on Newborn Birth Weight? A Systematic Review
Source: Nutrients. 2023 Mar 28;15(7):1649. doi: 10.3390/nu15071649 (PMC10096855; doi:10.3390/nu15071649)
Supplement: Supplementary file 1 [file nutrients-15-01649-s001.zip › Table S1.pdf]

Table S1. Characteristics of included studies, ordered by year of publication.

Study	Study period	Country	Setting	Study design
Godfrey et al., 1996 [17]	NR	United Kingdom	Midwives' booking clinic at the Princess Anne Maternity Hospital, Southampton	Prospective cohort; Single-center study
Mathews et al., 1999 [18]	May, 1994 to February 1996	United Kingdom	Antenatal booking clinics at St Mary's Hospital, Portsmouth, and its annexes	Prospective cohort; Multicenter study
Langley-Evans et al., 2003 [19]	January, 2001 to unknown end date	United Kingdom	Antenatal ultrasound dating scan clinic at Northampton General Hospital	Prospective cohort; Single-center study
Lagiou et al., 2004 [20]	March, 1994 to October, 1995	United States	Antenatal clinic at University Hospital in Boston	Prospective cohort; Single-center study
Moore et al., 2004 [21]	October, 1998 to April, 2000	Australia	An antenatal clinic at a public hospital and through the offices of 3 privately practicing obstetricians in Adelaide	Prospective cohort; Multicenter study
Denguezli et al., 2009 [22]	October, 2002 to February, 2003	Tunisia	Department of Obstetrics and Gynaecology. University Hospital Fattouma Bourguiba, Monastir	Retrospective case-control; Single-center study
Bawadi et al., 2010 [23]	NR	Jordan	Women's hospital, Northern Jordan	Retrospective cross-sectional, Single-center study
Watson and McDonald, 2010 [24]	NR	New Zealand	Maternal clinics, Northern New Zealand	Prospective cohort; Multicenter study
Crume et al., 2016 [25]	Unknown start date to November, 2013	United States	Prenatal, obstetric clinics at the University of Colorado Hospital	Prospective cohort; Single-center study
Diemert et al., 2016 [26]	2011 to 2013	Germany	University Medical Centre Hamburg, Eppendorf	Prospective cohort; Single-center study
Pathirathna et al., 2017 [27]	October, 2015 to June, 2016	Sri Lanka	Antenatal clinics at Teaching Hospital, Kurunegala	Prospective cohort; Single-center study
Grandy et al., 2018 [28]	July, 2012 to August, 2013	United States	Obstetrics Clinic, Oregon Health and Science University	Prospective cohort; Single-center study
Hjertholm et al., 2018 [29]	August, 2013 to September, 2013	Malawi	Nankumba Traditional Authority of Mangochi District	Prospective cross-sectional; Cluster study
Sharma et al., 2018 [30]	September, 2003 to June, 2006	United Kingdom	Two large teaching hospitals in Leeds	Prospective cohort; Multicenter study
Amezcua-Prieto et al., 2019 [31]	May, 2012 to July, 2015	Spain	Five hospitals of Eastern Andalusia	Retrospective matched case-control; Multicenter study
Eshak et al., 2020 [32]	2011 to 2014	Japan	Local governmental offices that issue pregnancy and child follow-up handbooks and/or obstetric facilities in fifteen Japanese communities	Prospective cohort; Multicenter study
Minato-Inokawa et al., 2020 [33]	May, 2015 to July, 2017	Japan	Department of Obstetrics and Gynecology, National Hospital Organization Kyoto Medical Center, Japan	Prospective cohort; Single-center study

NR: not reported
