# Supplementary material for: Is Maternal Carbohydrate Intake Having an Impact on Newborn Birth Weight? A Systematic Review
Source: Nutrients. 2023 Mar 28;15(7):1649. doi: 10.3390/nu15071649 (PMC10096855; doi:10.3390/nu15071649)
Supplement: Supplementary file 1 [file nutrients-15-01649-s001.zip › Table S2.pdf]

**Table S2.** Characteristics of study participants.

| Study | Sample size (Maternal and newborn pairs) | Maternal characteristics | | | | | | Newborn characteristics | |
|---|---|---|---|---|---|---|---|---|---|
| | | Overall characteristics | Age (years)[a] | Pre-pregnancy BMI ($kg/m^2$) [a] | Primiparous (%) | Smoked during pregnancy (%) | Alcohol consumption (%) | Birth weight (g) [a] | Gestational age (weeks) [a] |
| Godfrey et al., 1996 [17] | 538 | Caucasian women aged 16 years or older with singleton pregnancies | 26.4 ± 4.9 | 23.1 ± 4.4 | 53.0 | 25.8 | NR | Male: 3527 ± 496; Female: 3344 ± 463 | 40.2 ± 1.3 |
| Mathews et al., 1999 [18] | 693 | Nulliparous Caucasian women with singleton pregnancies | 25.8 ± 4.9 | 23.2 ± 3.9 | NR | 40.7 | NR | Male: 3425 ± 480; Female: 3281 ± 433 | Male: 40.1 ± 1.4; Female: 40.2 ± 1.3 |
| Langley-Evans et al., 2003 [19] | 226 | Healthy Caucasian women | 27.9 ± 5.2 | 25.4 ± 5.2 | NR | 16.9 | NR | Male: 3547 ± 459; Female: 3403 ± 517 | Male: 40.1 ± 1.0; Female: 40 ± 1.3 |
| Lagiou et al., 2004 [20] | 224 | Caucasian women aged less than 40 years and have a parity of no more than two | 18-24 y: 2.2%; 25-29 y: 27.7%; 30-34 y: 61.6%; 35+ y: 8.5% | <18 $kg/m^2$: 11.6%; 19-21 $kg/m^2$: 48.7%; 22-24 $kg/m^2$: 26.3%; >25 $kg/m^2$: 13.4% | 61.6 | 4.9 | 2.7 | Male: 3629 ± SE 40, Female: 3498 ± SE 48 | NR |
| Moore et al., 2004 [21] | 556 | Caucasian women aged 18 years or above with singleton pregnancy in which conception occurred without treatment for infertility | 29.0 ± 5.0 | 24.9 ± 5.7 | 33.6 | 19.6 | None of the women reported consuming > 2 drinks/ day (% NR). | Male: 3500 ± 472; Female: 3349 ± 574 | Male: 39.5 ± 1.5; Female: 39.4 ± 1.8 |

| | | | | | | | | |
|---|---|---|---|---|---|---|---|---|
| Denguezli et al., 2009 [22] | 350 (52 CAs, 298 COs) | Tunisian pregnant women with singleton pregnancies who delivered at term. CAs- women who delivered a newborn with a birth weight ≥ 4000 g; COs- women who delivered a newborn with normal birth weight | CAs,: 29.9 ± 4.6; COs: 28.7 ± 5.5 | CAs,: 28.2 ± 5.3; COs: 25.5 ± 4.2 | CAs,: 17.3; COs: 30.9 | NR | NR | NR | CAs: 40.4 ± 1.2; COs: 39.5 ± 1.4 |
| Bawadi et al., 2010 [23] | 700 | Healthy women of 18 years or older who had completed vaginal labor 24 to 48 hours | 28.7 ± SE 15.7 | 24.2 ± SE 0.2 | 24 | NR | NR | 3200 ± SE 20 | 36.7 ± SE 0.2 |
| Watson and McDonald, 2010 [24] | 439 | Healthy European and Polynesian women with a singleton pregnancy | 31.2 ± 5.1 | NR | NR | 11.4 | NR | 3551 ± 544 | NR |
| Crume et al., 2016 [25] | 1040 | Healthy pregnant women | 27.9 ± 6.1 | 25.5 ± 6.1 | 64.1 | 8.7 | NR | 3283.2 ± 432.5 | 39.6 ± 1.1 |
| Diemert et al., 2016 [26] | 200 | Healthy low-risk women of age 18 or older with singleton, spontaneously conceived pregnancy | 31.0 ± 3.5 | 24.7 ± 4.6 | 57 [b] | - | - | 3457.0 ± 497.0 | 40.0 ± 1.5 |
| Pathirathna et al., 2017 [27] | 126 | Healthy women with a singleton pregnancy | 28.8 ± 6.2 | 22.1 ± 4.3 | 35.7 | NR | NR | 2874.6 ± 497.0 | 38.8 ± 1.5 |
| Grandy et al., 2018 [28] | 41 | Healthy women with a singleton pregnancy | 30.9 ± 5.8 | 29.7 ± 7.1 | 59 | NR | NR | 3500 ± 400 | NR |
| Hjertholm et al., 2018 [29] | 132 | Pregnant women | NR | NR | NR | NR | NR | 3,104 ± 401 | NR |
| Sharma et al., 2018 [30] | 1196 | Low-risk pregnant women | 30 ± 5 | 25 ± 5 | 42 | FT: 17; ST: 12 | FT: 0.5 ± 0.9 [c]; ST: 0.2 ± 0.4 [c] | 3434 ± 559 | 40.0 ± 2.0 |
| Amezcua-Prieto et al., 2019 [31] | 1036 (518 CAs, 518 COs) | CAs- women who delivered a single live SGA newborn with no congenital malformations; COs- women who delivered a single live AGA newborn with no congenital malformations | NR | CAs: 23.1 ± 4.5; COs: 23.9 ± 4.1 | CAs: 63.9; COs: 57.5 | CAs: 28.8; COs:15.4 | CAs: 18.5; COs: 15.2 | NR | NR |

| | | | | | | | | | |
|---|---|---|---|---|---|---|---|---|---|
| Eshak et al., 2020 [32] | 78793 | Women with a singleton pregnancy | 31.0 ± 5.0 | <18.5 kg/m$^2$: 16.2%; 18.5-<25 kg/m$^2$: 73.8%; 25-<30 kg/m$^2$: 7.8%; ≥30 kg/m$^2$: 2.2% | 39.6 | 4.3 | 2.7 | NR | 38.9 ± 1.5 |
| Minato-Inokawa et al., 2020 [33] | 171 | Pregnant women aged 20 years or above | SGA group: 33.7±5.8; AGA group: 31.3 ± 5.3; LGA group:32.7 ± 6.5 | SGA group: 20.7 ± 3.1; AGA group: 21.2 ± 3.1; LGA group:21.6 ± 3.1 | SGA group: 41.2; AGA Group: 44.4; LGA group: 40.0 | NR | NR | SGA group: 2489 ± 217; AGA group: 2966 ± 285; LGA group: 3589 ± 356 | SGA group: 39.1 ± 1.1; AGA group: 38.8 ± 1.2; LGA group: 38.8 ± 1.8 |

[a] Mean ± Standard deviation if not mentioned otherwise. [b] Primi-gravida. [c] units/day. AGA: appropriate-for-gestational-age; BMI: body mass index; CAs: cases; COs: controls; FT: first-trimester; LGA: large-for-gestational-age; NR: not reported; SGA: small-for-gestational-age; SE: standard error of mean; ST: second-trimester.
